# Physical Training in the Public Schools of Atlanta

**Published:** 1903-03

**Authors:** Theodore Toepel

**Affiliations:** Physical Director Public Schools, Atlanta, Ga.


ATLANTA

### Journal-Record of Medicine.

**Successor to Atlanta Medical and Surgical Journal, Established 1855, and Southern Medical Record, Established 1870.**

Vol. IV. MARCH, 1903. No. 12.

BERNARD WOLFF, M.D., EDITOR, Nos. 319-20 Prudential.

Published Monthly.

M. B. HUTCHINS, M.D., BUSINESS MANAGER, No. 64 Marietta St.

### ORIGINAL COMMUNICATIONS.

### PHYSICAL TRAINING IN THE PUBLIC SCHOOLS OF ATLANTA.

By THEODORE TOEPEL, M.D.,
Physical Director Public Schools, Atlanta, Ga.

It is with great pleasure that I take this opportunity to address you on a subject which upon first thought may seem to some of you peculiar in its selection, as being one of no particular interest to an assemblage of physicians, but, before I have concluded, I hope to have shown you that the subject before me bears close relation to the medical profession, and, as it is the physician's care to promote the physical welfare of the community, I trust that my little talk on Physical Training in the Atlanta public schools to-night may prove well-chosen.

Education, as you know, involves the development of the physical, moral and mental powers with which the human being is endowed. I mention the physical first, as you notice, because nature's process should not be reversed, as is so often seen where the unfolding of the mental faculties has been accomplished at the expense of the physical. The laws governing physical and mental capacities must conform to the principles of physiology, psychology and pedagogy.

Physiology teaches us that the action of the organs of circulation, secretion and respiration is accelerated by physical exercise. It is a vital law that every organ in activity draws toward it a greater quantity of nutrient fluid than when in a state of repose. There is an active congestion of all the organs during physical exercise, hence more active performance of function and a marked increase in the nutritive forces during the period of activity. All the organs and all the tissues of the body are the seat of a more active circulation, and we know that the nutrition of an organ is in proportion to the quantity of blood which passes through it. Under the influence of the greater quantity of blood which traverses the lung, this organ becomes more active and introduces into the system a greater quantity of air. Exercise has a stimulating action on all the organic functions because it renders the circulation in all the organs more active. The blood makes all parts of the system share in the stimulation which the will has sent to the muscles to put them into action, and this stimulation is the more marked because the blood is warmed by the friction against the walls of the vessels through which it passes more rapidly. Thus when the limbs move, the internal organs cannot remain inert, and the whole organism performs its functions with more energy under the influence of muscular contraction.

Perhaps the most interesting effect of this active congestion of the organs under the influence of bodily exercise is that experienced by the brain. All thinkers have noticed that physical exercise is favorable to brain-work. It renews the impoverished brain cells, furnishing them with blood saturated with oxygen. We know that when a muscle is supplied with good blood and is stimulated to action it grows. The nerve centers of the brain which stimulate the muscle and initiate its action are affected at the same time. These nerve centers act on future occasions with more exactness and rapidity when the same exercise is carried out.

Statistics of experiments made on pupils at schools, on inmates of reformatories and insane asylums prove the wonderful effect of physical exercise in brain development, showing that the mental and physical equilibrium was thereby restored and proving again the truth of the old quotation, "*Mens sana in corpore sano.*"

Physical exercise forms a part of the school curriculum mainly because it counteracts the fatigue resulting from mental exertion, creating renewed energy, thus rendering pupils better able to withstand the mental strain accompanying school life. It builds up weak and poorly developed pupils and corrects slight deformities which are otherwise overlooked by parents and teachers. Of course the cure of graver deformities is the work of the specialist who is able to render the individual treatment which the case may demand. Physical training was introduced into the public schools of Atlanta in September in the year 1899, and has since been continued as a branch of the regular curriculum. As conducted now the children exercise fifteen minutes daily, the lower grades in three periods of five minutes each and the upper grades in two periods of eight and seven minutes each. At the high schools ten minutes per day are devoted to the work. Teachers receive instructions once every month and have the exercise in pamphlet form for their convenience. The lessons are conducted in the class-rooms, which are as thoroughly ventilated as conditions permit before the exercises are given. Occasionally the pupils in the upper grades exercise on the playgrounds, eight of which are furnished with outdoor gymnasiums. These consist of horizontal bars, flying rings, climbing ropes and poles for boys, rings, balancing beams and seesaws for girls. The money required to purchase this apparatus was made at the field-day exercises. These outdoor gymnasiums are used by the children during recess.

With the limited time given to this branch the results in physical education have been satisfactory. The children love the work and have improved noticeably, their carriage being better in standing, walking and sitting. The good results are to a great extent due to the conscientious efforts of the teachers who make good use of the allotted time, which nevertheless is insufficient to accomplish what might be done could more time be devoted to the work.

Physical training must be progressive in order that it may conform to the growth of the body. Exercise in the schoolroom between the desks fulfills this requirement only partially. The mind is relieved for the time, being occupied with work of a wholly different nature; the body is allowed to stretch and assume more healthful attitudes, but this is not sufficient. Just as little as the mind is satisfied with an incomplete knowledge of reading, arithmetic, geography, history, etc., so does the body require progressive work as it advances in development. From year to year should there be increased variety and difficulty, exercises of swiftness should be followed by those requiring endurance, strength and dexterity, culminating in the higher grades in various branches of gymnastics and athletics. Games and plays should always form a prominent feature for every age. This means an adequate space for exercise in every school, a closed hall for the winter and an open playground or athletic field for the summer.

These are ideal conditions, and to attain them it is necessary to repeatedly call the authorities' attention to these needs until they are effected. The complete equipments found in European schools for this work show the possibility for these ideal conditions to exist in this country as well. The amount of money available in the United States for the education of its school children exceeds that of any other country in the world; hence the financial aspect of the question ought not to offer an obstacle to the solution of the problem, especially if we consider the value of physical exercise as a hygienic and prophylactic agent, not to mention its educational and ethical value in aiding to rear a generation of strong self-controlling men and women.

To do full justice to the children as well as to the work a teacher should not have more than five hundred children under his direct supervision, and not twelve thousand as is the case in our schools at the present time. Then an anthropometrical examination of each child could be made at the beginning and at the close of the school year. I consider it essential that the child's physical growth be watched, measured and recorded just as regularly as its mental development. Instead of fifteen minutes, thirty minutes daily should be devoted to the work. This is proportionately little enough when we consider that the children spend five hours a day at school.

Montaigne well says in speaking of a man as he should be: "I would have the disposition of his limbs formed at the same time with his mind. 'T is not a soul, 't is not a body we are training, but a man, and we must not divide him."

Col. Francis W. Parker, one of the foremost thinkers and educators of to-day, has said: "It may never be known scientifically what a tremenduous influence the body and all its organs, every nerve and muscle, vein and artery, exert upon the brain and consequently upon the intellect, and the more I see of the good work of physical culture in the school the more I believe in it; the more I study psychology, especially physiological psychology, the stronger my belief becomes in physical training."

Dr. DuBois Reymond, the physiologist, says: "Man is adapted to self-improvement by means of exercise; it makes his muscles stronger and more enduring; his skin becomes fortified against all injury; through exercise his limbs become more flexible, his glands more productive; it fits his central nervous system for the most complicated functions; it sharpens the senses, and by it his mind reacting upon himself is enabled to augment its own elasticity and versatility."

It is not the purpose of physical training to make Sullivans, Jeffries and Roman gladiators of our children. Its right purpose reaches out to one aim, the perfect development of physical power, to help make complete the work of true education which strives to attain the harmonious development of all the faculties with which we are endowed.

In closing I ask the cooperation of the Atlanta Society of Medicine and of all who are interested in the subject in agitating the matter until more favorable conditions for the physical welfare of the school children of Atlanta be realized.

